# Covalent Cross-Linking as a Strategy to Prepare Water-Dispersible Chitosan Nanogels

**DOI:** 10.3390/polym15020434

**Published:** 2023-01-13

**Authors:** Sara Muñana-González, Antonio Veloso-Fernández, Leire Ruiz-Rubio, Leyre Pérez-Álvarez, José Luis Vilas-Vilela

**Affiliations:** 1 Grupo de Química Macromolecular (LABQUIMAC), Departamento de Química Física, Facultad de Ciencia y Tecnología, Universidad del País Vasco UPV/EHU, 48940 Leioa, Spain; 2 BCMaterials, Basque Center for Materials, Applications and Nanostructures, UPV/EHU Science Park, 48940 Leioa, Spain

**Keywords:** chitosan, polyethylene glycol diacid, nanogels, water-soluble, cross-linking

## Abstract

Due to the environmental problems generated by petroleum derivative polymers as mentioned in Agenda 2030, the use of natural polymers is increasing. Among them, cellulose and chitin are the most widespread biopolymers available in nature. Chitosan, obtained from chitin, is a really good candidate to develop nanocarriers due to its polyelectrolyte nature and ease of chemical modification. However, chitosan presents a solubility drawback in an aqueous medium at physiological pH (pH = 7.4), which restricts its applicability in biomedicine. In this work, nanogels were successfully synthesized from chitosan systems with different water solubilities (chitosan, oligosaccharide chitosan, and quaternized chitosan) using the reverse microemulsion method and polyethylene glycol diacid (PEGBCOOH) as a covalent cross-linking agent. Cross-linking with PEGBCOOH was analyzed by proton nuclear magnetic resonance (^1^H-NMR), which allowed for nanogels to be prepared whose size and swelling were comparatively studied by transmission electron microscopy (TEM) and dynamic light scattering (DLS) and zeta potential, respectively. The particle size of the swollen nanogels showed a different pH-responsive behavior that decreased for chitosan, increased for oligosaccharide chitosan, and remained constant for quaternized chitosan. Nevertheless, a drastic reduction was observed in all cases in the culture medium. Along the same line, the dispersibility of the synthesized nanogels in different media was comparatively evaluated, showing similar values for the nanogels prepared from soluble chitosans than for water insoluble chitosan as a consequence of the cross-linking with PEGBCOOH. After 6 months of storage of the dried nanogels, the water dispersibility values remained constant in all cases, demonstrating the stabilizing effect of the employed cross-linking agent and the potential use of synthesized nanogels as substrates for drug delivery.

## 1. Introduction

In recent years, natural polymers have been used for a wide variety of biomedical applications due to their similarity with the extracellular matrix, mechanical compatibility, high biocompatibility, and high water retention capacity [1]. Indeed, because of the non-toxicity of their degradation products, they have a promising role in applications such as targeted delivery and nutrient, enzyme, and cell encapsulation [2].

It is particularly interesting to bring these materials of natural origin into the field of nanotechnology in order to take advantage of the unique properties that come with the reduced particle size. Used as so called “nanocarriers”, biopolymers protect the cargo from external disturbances and the effects of the environment that could negatively affect their performance [3,4]. Given their limited impact on natural environments, the applicability of polymeric nanocarriers extends even further than the area of biomedicine, having special relevance in agriculture or environmental technologies [5]. This also aligns well with the general interest in using natural biomass as crude resources for chemical, polymer, and material development.

Regarding polymers derived from natural biomass, after cellulose, chitin is the most widespread biopolymer available in nature, being primarily found in crustaceans, insects, and microorganisms. By performing alkaline deacetylation of chitin, the polymeric derivative chitosan is obtained. Chitosan is a linear polysaccharide formed by the union of units of β-(1-4)-2-acetamido-D-glucose (GlcAC) and β-(1-4)-2-amino-D-glucose (GlcN) [6]. Protonation of these amino groups converts chitosan in a polyeletrolyte, giving rise to changes in its structure and properties, which are caused by the cationic structure acquired by its protonation at values of pH below its pKa of 6.3–6.7 [7].

The various biological properties of chitosan that are interesting in the area of biomedicine should be highlighted [8]. Among them, its biodegradability, biocompatibility, sustainability, and mucoadhesion. In addition, chitosan promotes adhesion, cell proliferation, and differentiation, has a broad antimicrobial effect, does not cause an immune reaction, and is not carcinogenic [9]. However, chitosan has a drawback in terms of its solubility in an aqueous medium at physiological pH (pH = 7.4), since it is only soluble in an acid medium, restricting its use in biomedicine. When the pH values are higher than 6.5, the amino groups are in a neutral state, causing the appearance of intra- and inter-molecular hydrogen bonds. These strong interactions, along with others of hydrophobic nature, give place to crystalline domains, which make chitosan not soluble in most neutral and basic solvents [10]. In recent years, numerous studies have been carried out to provide an answer for the chitosan solubility problem, mostly focusing on two main strategies: the reduction in the molecular weight of chitosan because it has been observed that chitosan oligomers, which can be obtained by degradation of chitosan, are soluble in water, and its chemical modification via methods such as carboxymethylation, acetylation, quaternization, “PEG-ylation”, and sulfonation [11,12]. Among these chemical modifications, it is worth mentioning the chitosan quaternization process from the reaction between glycidyl trimethyl ammonium chloride (GTMAC) and chitosan by the nucleophilic substitution of the primary amino group in chitosan [13,14]. This improves the water solubility of the polymer by introducing a permanent positive charge in the structure. Quaternized chitosan has been widely studied as it has a wide application in the environmental field such as waste treatment because it is a good absorbent and flocculent agent, and in the biomedical field due to its good biocompatibility, biodegradation, biological, antimicrobial, and low toxicity [14,15].

One of the main applications of chitosan and its derivatives in the biomedical field is that related to the controlled release of active substances. For this, chitosan is typically modified in the form of a hydrogel. The cationic nature of chitosan allows for the formation of physical hydrogels through electrostatic cross-linking that in diluted conditions leads to nanometric dimensions. Chitosan is also able to self-aggregate and form nanoparticles in an aqueous medium due to hydrophobic interactions [16], and by varying the number of hydrophobic groups of chitosan chains, it is possible to control their particle size [17]. However, and due to the insoluble nature of chitosan, nanoparticles prepared by both methods rapidly precipitate at physiological pH [18].

The presence of reactive groups in chitosan also makes it prone to being modified to generate networks by covalently bonding with cross-linking agents. The chemical cross-linking of chitosan chains allows for the free diffusion of water or bioactive materials and improves the mechanical properties [12]. The most common cross-linking agents for chitosan are aldehydes [19] and epoxides [20], among others [21], highlighting especially glutaraldehyde. However, due to the demonstrated toxic character of this compound [22], there is a current need to find new biocompatible cross-linking agents for the preparation of chitosan nets.

Polyethylene glycol diacid (PEGBCOOH) is a cross-linking agent that has been scarcely used in the literature [23,24], which could add an interesting effect that increases the dispersibility of chitosan nanometric hydrogels. PEG is a neutral, non-toxic, and biodegradable water-soluble polymer. It is a synthetic polymer present in a wide variety of foods, cosmetics, personal care products, and pharmaceuticals, and is typically used in a wide range of biomedical applications to increase nanoparticle stability [25]. Mincheva et al. [24] proposed a method to prepare chitosan macroscopic hydrogels using polyethylene glycol diacid (PEGBCOOH) as a cross-linking agent, however, they did not restrict this cross-linking within the nanoscale.

Combined with different techniques in which the particle size is controlled and restricted to colloidal dimensions, the cross-linking of chitosan can be used to produce hydrogels at the microscopic and nanometric scale. To this end, heterogeneous crosslinking reactions are of great interest because reactions are carried out in a nanometric confined space.

Therefore, various heterogeneous methods have been distinguished such as emulsion and microemulsion or reverse emulsion and microemulsion reactions, in addition to different dispersion and precipitation methods. In this work, the reverse microemulsion method was carried out because it is considered as one of the most effective in producing homogeneous nanoparticle size distributions. Within the reverse micelles of nanometer size, reactions of polymerization/cross-linking can be carried out, acting as nanoreactors, which allows for nanosized hydrogels (nanogels) to be obtained [26,27].

Surface modifications by PEG of nano-sized chitosan particles are arousing great interest for therapeutic applications due to their potential, since they increase the physical stability of the particles and prolong the time of circulation in blood, reducing the probability of being eliminated by the reticuloendothelial system [28]. As expected, crosslinking between PEGBCOOH and chitosan in a microemulsion was revealed as an effective way to prepare chitosan nanogels [29]. However, the role of this cross-linking agent to promote stable and water-dispersible nanogels has not been evaluated yet. Thus, taking all of the above into account, PEGBCOOH was here explored as a cross-linking agent for various chitosan systems (chitosan, oligosaccharide chitosan, and quaternized chitosan) with different water solubilities to analyze its influence on the water solubility of the final nanogels. Studies on the particle size distribution, relative charge, and structure coupled with the evaluation of dispersibility in different media were carried out. This work aims to demonstrate the potential use of synthesized cross-linked chitosan nanogels in biomedicine as a substrate for drug delivery systems.

## 2. Materials and Methods

### 2.1. Materials

Low molecular weight chitosan (50,000–190,000 g/mol) and chitosan oligosaccharide (5000 g/mol) were purchased by Aldrich (St. Louis, MO, USA). Quaternized chitosan was obtained from low molecular weight chitosan, as described below by reacting commercial chitosan with glycidyltrimethyl ammonium chloride (GTMAC) [14]. Poly(ethylene glycol)bis(carboxymethyl)ether (PEGBCOOH, 600 g/mol), 1-(3-dimethylaminopropyl)-3-ethylcarbodiimide hydrochloride (EDC, Sigma-Aldrich, St. Louis, MO, USA), N-hydroxysuccinimide (NHS, Sigma-Aldrich, St. Louis, MO, USA), NaOH 2 N (Panreac, Barcelona, Spain), N-tert-butoxycarbonyl-2,2′-(ethylenedioxy)bis(ethylamine), and HCl (Panreac, Barcelona, Spain, 0.5 N) were employed for the cross-linking reactions.

In the preparation of the microemulsions and purification of the nanoparticles obtained, the following were used: Triton X-100 (Aldrich, St. Louis, MO, USA), cyclohexane (for synthesis, Panreac, Barcelona, Spain, 99.5%), hexanol (for synthesis, Aldrich, St. Louis, MO, USA, 98%), acetic acid (for analysis, Panreac, Barcelona, Spain, 99.8%), NaOH (s) (for analysis, Panreac, Barcelona, Spain, 98%), and ethanol (for analysis, Panreac, Barcelona, Spain, 96%).

For ^1^H-NMR analysis, the CDCl_3_ and D_2_O solvents were used.

DMEM F:12 PIS culture medium (Gibco, ThermoFisher, Waltham, MA, USA), whose main composition is L-alanil-L-glutamine (2.50 mM), anhydrous calcium chloride (1.05 mM), potassium chloride (4.16 mM), sodium bicarbonate (29.02 mM), sodium chloride (120.61 mM), and D-glucose (17.51 mm), was also employed.

### 2.2. Synthesis of Quaternized Chitosan

Low molecular weight chitosan (2.0 g) and perchloric acid (1.9 g, 1.14 mL) were dissolved in 30 mL of distilled water at room temperature (~20 °C). GTMAC was separately dissolved (5 g, 4.47 mL) in 20 mL of distilled water. Subsequently, the prepared solution of GTMAC was added over the chitosan solution in three aliquots at intervals of 30 min with an increase in the temperature to 60 °C. Once the third aliquot was added, the reaction mixture remained at 80 °C for 4 h. Afterward, the yellowish solution was poured into acetone to precipitate the obtained product. After washing several times with acetone, the obtained product was collected by vacuum filtration. In order to obtain a higher purity of the quaternized chitosan, the product was dissolved again in water and kept in magnetic agitation until the total redissolution of the solid. Once it was completely dissolved, the product was precipitated into acetone and vacuum filtering. Subsequently, quaternized chitosan was dried at 60 °C for 48 h.

### 2.3. Synthesis of Chitosan Nanogels with PEGBCOOH

The cross-linking agent PEGBCOOH was activated, obtaining N-hydroxysuccinimide esters from their carboxylic acid groups. To do this, NHS (220 mg, 1.9 mmol) was dissolved in 20 mL of distilled water at room temperature. Once the solid was completely dissolved, PEGBCOOH (500 mg) was added, keeping the pH value constant to 5.4, using a 2 M NaOH solution. After 15 min of stirring, EDC (750 mg, 3.91 mmol) was added slowly, keeping the pH value constant, using a 2 M HCl solution. The reaction mixture was kept at constant stirring at ambient temperature for 4 h. In this way, the molar relationship established by the reagents used (PEGBCOOH:NHS:EDC) was 2:4:10.

After carrying out the activation of the cross-linking agent, the cross-linking reaction between PEGCOOH and chitosan took place at room temperature. To carry out at the nanometer scale, the reverse microemulsion technique was followed. To this end, two reverse microemulsions were prepared (one for the cross-linking agent and the other one for chitosan). Cyclohexane (50 mL) and 1-hexanol (20 mL) were mixed in two different reactors. In one of the solutions, 20 mL of the solution containing the activated cross-linking agent was added, and in the chitosan microemulsion, 20 mL of a solution at 1% (1 g in 100 mL) of the corresponding chitosan previously prepared in 1% acetic acid. In both mixtures, the surfactant Triton-X was slowly added until the mixtures became transparent (microemulsion). After 1 h of constant magnetic stirring, the solution with the cross-linking agent was slowly poured over the one containing the chitosan. The resulting reverse microemulsion was maintained at constant magnetic agitation for 24 h. After this period, the precipitation of the nanogels was carried out by adding the same amount of EtOH volume as the total volume of the microemulsion. After decanting the nanogels, they were purified by performing several cycles of centrifugation (6400 rpm, 15 °C, 15 min) and cleaning with EtOH. Finally, the nanogels were dispersed in water, subjected to dialysis in water for a week, and dried.

## 3. Characterization of the Nanogels

### 3.1. Proton Nuclear Magnetic Resonance (^1^H-NMR)

^1^H NMR spectra were performed for each of the pristine chitosans and for each of the prepared nanogels. Referring to the sample preparation, all chitosan nanogels as well as oligosaccharide and quaternized chitosan were dispersed with a concentration of 10 mg/mL in deuterated water (D_2_O). In the case of the pristine low molecular weight chitosan, it was dispersed in 10 mg/mL concentration in an aqueous solution of deuterated acetic acid 1%, also in deuterated water. In each case, chemical displacements (δ) were measured in ppm in relation to deuterated water (D_2_O) (δ = 4.8 ppm).

### 3.2. Determination of Free Amino Groups Percentage in Quaternized Chitosan

A colorimetric method was used for the quantitative determination of the concentration of free amino groups in the quaternized chitosan. For this, 8 mL of quaternized chitosan solution in buffer acetic/acetate buffer (4 M, pH = 5.2) was prepared (0.009 g/mL) by stirring at 20 °C for 24 h. Subsequently, 1.5 mL of the chitosan solutions was taken and mixed with 1 mL of ninhydrin. The sample was kept in reflux for 30 min at 109 °C. Afterward, the solution was cooled and diluted to 50 mL using a solution of EtOH:H_2_O at 50:50. Finally, its absorbance was measured on a UV/Vis spectrophotometer at 570 nm. According to the previous calibration curve (y = 8449222.1278x + 0.0992, R^2^ = 0.9939) carried out with modified ninhydrin for chitosan, the content in the amino groups in the chitosan samples after quaternization was determined.

### 3.3. Transmission Electron Microscopy (TEM)

Microphotographs of the nanogels were obtained with a Philips CM120 Biofilter transmission electron microscope (TEM).

For sample preparation, chitosan nanogels were dispersed in an aqueous solution in a 1 mg/mL concentration, and kept under constant magnetic agitation for 3 days. Once the stirring time was over, a drop of the dispersion was deposited on a carbon fiber grid and dried by glow discharge.

### 3.4. Dynamic Light Scattering (DLS) and Zeta Potential

To determine the particle size (z-average and the polydispersity index, PDI) of the synthesized nanogels, the dynamic light scattering (DLS) technique was used. Simultaneously, measurements of Z potential (ζ) were carried out to study the distribution of the surface charge at the solid/water interface.

Both measurements were made using a Zetasizer Nano Z equipment (Malverns Instruments Ltd.) at room temperature using a 1 cm wide bucket (Malvern Instruments DTS1070). For sample preparation, the chitosan nanogels were dispersed in an aqueous solution (1 mg/mL). Each sample was diluted 10 times to prevent agglomerates and constant magnetic agitation was maintained for 48 h. To adjust the pH of the medium to the physiological pH value, the pH of each aliquot was modified using a 0.1 M NaOH solution.

### 3.5. Dispersibility of Nanogels

The dispersibility of the nanogels was estimated following the method described in the literature [30]. To this, three different dispersions were prepared in water at pH = 6.0 (adjusting the pH by adding dropwise a solution of 0.1 M acetic acid), pH = 7.4 (adjusting the pH by adding dropwise a solution of 0.1 M NaOH), and the culture medium (DMEM F:12 PIS). The initial mass of the nanogel sample (~5 mg) was accurately weighed and dispersed in 2 mL of each medium by magnetic stirring (3000 rpm) for 1 h at room temperature. When the solution was clear, increasing mass of the sample was added until the lack of transparency or the presence of aggregates was observed by the naked eye. The reported data are the average of three measurements.

## 4. Results and Discussion

### 4.1. Synthesis of Quaternized Chitosan

**Figure 1:**
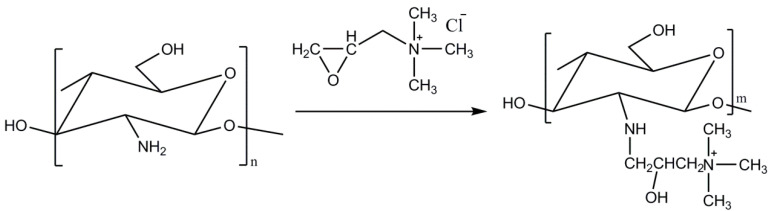
Synthesis of the water soluble N-[(2-hydroxy-3-trimethylammonium) propyl] chitosan.

Quaternized chitosan was synthesized from low molecular weight chitosan (Figure 1) to obtain water soluble chitosan in an aqueous medium at physiological pH. This water solubility is explained by the incorporation of the positive charge characteristic of the quaternary ammonium cation that enhances the solubility, regardless of the pH of the aqueous medium due to the electrostatic repulsion of the chitosan chains. Although different approaches have been explored to prepare quaternized chitosan derivatives such as grafting with [(acryloyloxy)ethyl]trimethylammonium chloride [31], the reaction with glycidyltrimellthylammonium chloride, specifically in the presence of perchloric acid, is one of the most exploited synthetic procedures in the literature, which leads to obtaining the N-[(2-hydroxy-3-trimethylammonium) propyl] chitosan.

**Figure 2:**
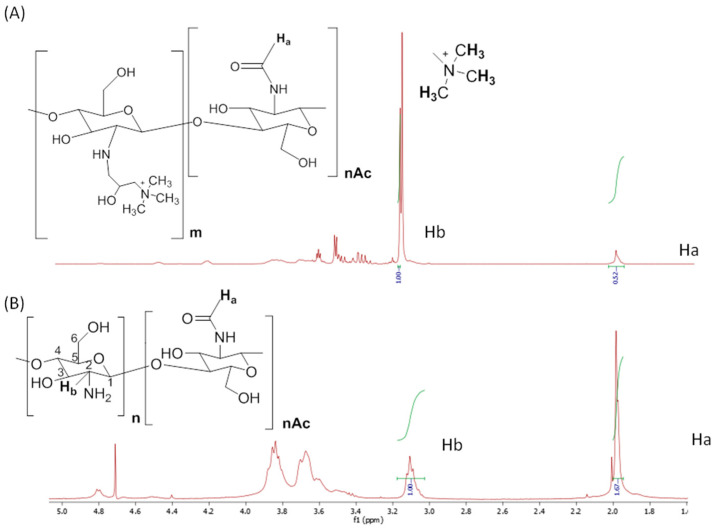
^1^H NMR spectrum of the (**A**) chitosan and (**B**) synthesized quaternized chitosan.

^1^H NMR spectroscopy (Figure 2) demonstrated the successful quaternization reaction of chitosan. Regarding pristine chitosan, Figure 2A shows the typical ^1^H NMR spectrum of chitosan, where the signal of 2.0 ppm corresponded to the three methyl protons of the N-acetylglucosamine residues (GlcNAc, Ha) from the deacetylation of chitin, and the signal that appeared at 3.12 ppm represents the proton H-2 of glucosamine (GlcNH_2_, Hb), which indicates the percentage of primary amine. The H-1 resonance signal that appeared at 4.8 ppm overlapped with that of the solvent. The rest of the protons of chitosan (H3–H6) had similar electron densities and chemical shifts, so that in the spectrum of linear chitosan, their corresponding signals overlapped and produced a wide band that was observed between 3.5 and 4.0 ppm. The methyl protons of the –NH–CO–CH_3_ group (Ha) were selected as the reference for the calculation of the deacetylation degree (DD) of the initial chitosan by the comparative integration of the signal of the H-2 proton of the glucosamine moieties (Hb). A DD value of 63% was measured for low molecular weight chitosan. In the case of quaternized chitosan, a strong peak appearing at 3.18 ppm that was attributed to the nine protons of the quaternary ammonium group, –N^+^(CH_3_)_3_, could be clearly observed [32]. However, the overlap of this resonance signal with that of the unreacted H-2 protons restricts the quantitative analyses of the quaternization degree of chitosan.

The reaction of chitosans with ninhydrin is well-known as a rapid, sensitive, and reproducible method for the quantification of glucosamine (GlcNH_2_) units [33,34]. Accordingly, the concentration of N-acetyl glucosamine residues on the prepared quaternized chitosan was measured by this colorimetric test (Abs = 8.4 × 106 C (mol/mL) R^2^ = 0.9939) with the aim of quantifying the carried out chitosan functionalization reaction. The molar percentage of the remaining free amine moieties in the final chitosan was 3.2 ± 0.3, which led to a quaternization degree of 94%.

### 4.2. Synthesis of Chitosan/PEGBCOOH Nanogels

The cross-linking of the different chitosans (low molecular weight chitosan, oligosaccharide chitosan, and quaternized chitosan) was carried out using diacid polyethylene glycol (PEGBCOOH) as a covalent cross-linker and the reverse microemulsion technique as a method of restricting the particle size at the nanoscale. According to the literature, the incorporation of PEGBCOOH seems to contribute to the water dispersibility at pH = 7.4 (physiological pH) of the cross-linked chitosan nanogels. Indeed, different works have already been devoted to the synthesis of chitosan covalent nanogels by the direct amidation reaction between the amino groups of chitosan and the carboxylic groups of different di- and triacids including succinic acid, tartaric acid, and PEGBCOOH, among others, showing high water dispersibility at pH 6.5 [35]. Pujana et al. [29] carried out this amidation reaction specifically with tartaric acid and PEGBCOOH within the microemulsion medium and they displayed an increasing water dispersibility at neutral-basic pH with the carboxylic acid content (soluble at pH = 8 for PEGBCOOH >37.5% mol).

**Figure 3:**
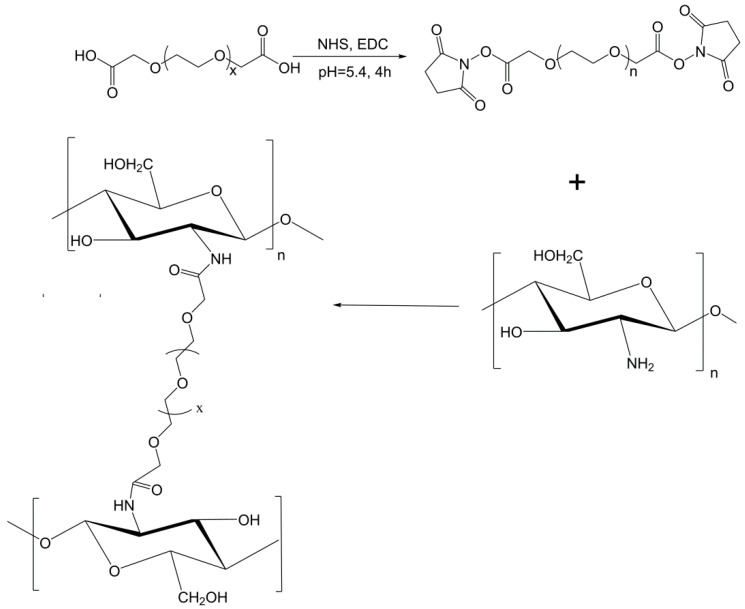
Cross-linking reaction between the activated PEGBCOOH and the glucosamine (GlcNH2) units (n) of the different chitosans.

As described in the experimental section, the cross-linking of chitosan with the flexible PEGBCOOH molecules requires the previous activation of their carboxylic groups, giving rise to the formation of intermediate N-hydroxysuccinimide esters (Figure 3). Subsequently, the cross-linking takes place by means of the nucleophilic attack of the free amine groups present in the different chitosans on the NHS-activated acid and with the formation of the consequent amide bond.

**Figure 4:**
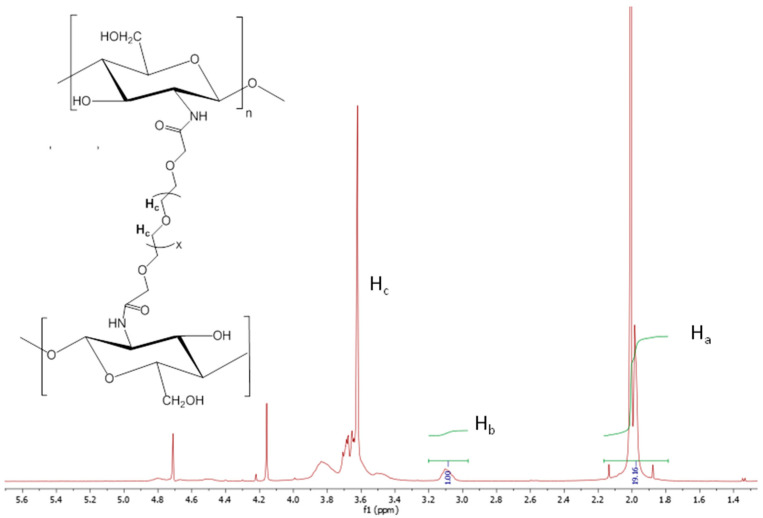
^1^HNMR spectrum of the low molecular weight chitosan nanogels obtained by the amidation reaction with PEGBCOOH in the reverse microemulsion.

**Figure 5:**
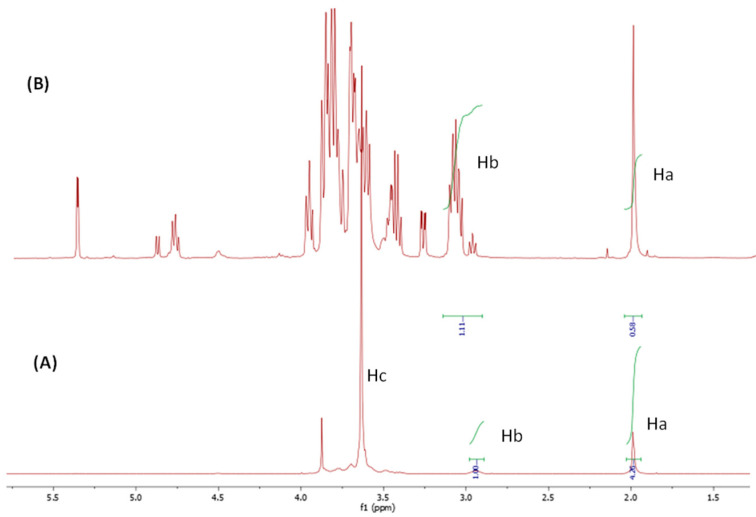
^1^HNMR spectrum of the (**A**) oligosaccharide chitosan nanogels and (**B**) unmodified oligosaccharide chitosan.

**Figure 6:**
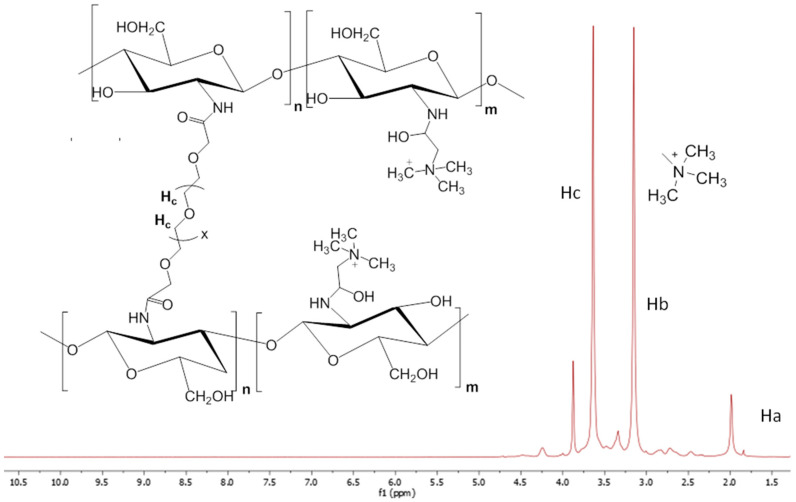
^1^HNMR spectrum of the quaternized chitosan nanogels.

The incorporation of PEG to the chitosan backbone was qualitatively verified in all of the cross-linking reactions (Figure 4, Figure 5 and Figure 6) by ^1^HNMR. The spectra of all of the nanogels showed a clear appearance of the strong signal of the alkyl protons (Hc) of the –O–CH_2_–CH_2_–O fragments of PEG at 3.5–3.8 ppm (Hc), even for quaternized chitosan (Figure 6), despite the initially low GlcN content (3%). In addition, a decrease in the characteristic signal of the free amine groups (GlcN, Hb) could be clearly observed in relation to that of the methyl protons of the acetyl group of N-acetamidoglucose units (Ha) compared with the corresponding unmodified chitosan. As expected, differences were also found when the ^1^HNMR spectra of the employed initial chitosans were compared. For example, the lower molecular weight of oligosaccharide chitosan is reflected in the high resolution of its spectrum in comparison with that of the polymeric low molecular weight chitosan. As described above, the presence of the quaternary ammonium group on the backbone of chitosan led to an easily recognizable sign at 3.18 ppm in its spectrum (Figure 6).

The incorporation of PEG to the chitosan backbone was not only qualitatively verified by ^1^H NMR. This technique also allowed, in turn, for the determination of the final composition of the nanogels synthesized from unmodified chitosan. In this sense, since the linkage of PEGBCOOH to the chitosan backbone takes place through the free amino groups, the variation in the relative intensity of the Ha integrals with respect to the signal of the Hb integrals, makes the estimation of the extent of the modification reaction possible, that is, the cross-linking for each chitosan type, except in the case of quaternized chitosan, which was due to the overlapping of the Ha peak with those of the protons of the –N^+^(CH_3_)_3_ methyl groups. In this way, the relative intensities of the named signals were compared in the spectra of the cited pristine chitosan (low molecular weight, oligosaccharide). Assuming that each PEGBCOOH molecule reacts with two GlcN units (2-amino-D-glucose) and there was no intramolecular cross-linking, the degree of cross-linking (%) can be defined as half the value of the degree of modification (% DM). For quaternized chitosan nanogels, the GlcN content in the nanogels, and thus, the cross-linking degree, was quantified by the ninhydrin test in comparison with that of the initially synthesized quaternized linear chitosan.

Table 1 summarizes the initial GlcN percentage (DA) determined as described above by ^1^HNMR or the ninhydrin test (quaternized chitosan) as well as the value calculated for GlcN after nanogel formation, which allowed for the estimation of the overall cross-linking degree in the different networks for the studied chitosan types (low molecular weight, oligosaccharide, and quaternized chitosan).

As expected, the high quaternization degree achieved in the chitosan modification reaction led to loosely cross-linked quaternized chitosan networks. Accordingly, unmodified chitosans led to higher modification grades, and greater cross-linking degrees were measured for oligosaccharide chitosan, according to the lower molecular weight of the initial chitosan, which seemed to enhance reactivity.

### 4.3. Morphology, Size, Swelling, and ζ-Potential of Chitosan Nanogels

**Figure 7:**
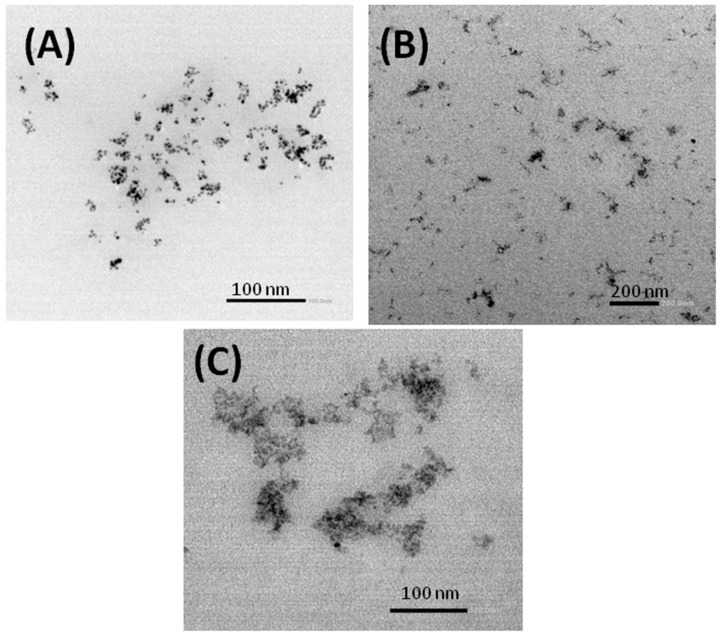
TEM micrographs of the synthesized nanoparticles: (**A**) chitosan (low molecular weight), (**B**) oligosaccharide chitosan, and (**C**) quaternized chitosan.

Different authors have reported effective control of the colloidal size of the chitosan nanogels by using the inverse microemulsion technique [36]. As previously explained, the inverse microemulsion is a transparent synthesis medium in which colloidal-sized droplets of the aqueous phase are dispersed in a continuous oily phase stabilized by surfactant molecules at the oil/water interface. Inside these nanometric droplets, the crosslinking reaction of the confined linear chitosan with a specific cross-linking agent, PEGBCOOH, takes place. Due to this, the size of the nanogels is limited and interparticle aggregation is reduced. The morphology and the size of the synthesized nanoparticles in the collapsed state were evaluated by TEM (Figure 7).

The hydrogels obtained presented a spherical morphology. However, the loss of sphericity in some of the nanoparticles can be attributed to the drying process, since the soft nature of hydrogels can reduce the spherical shape on drying [37]. It should be noted that the nanoparticles were synthesized from the chitosan of different molecular weights, but all of the nanoparticles showed the same characteristics, with similar diameters and spherical shapes, which validates the inverse microemulsion as a technique capable of conditioning the particle size at the nanometer scale.

**Figure 8:**
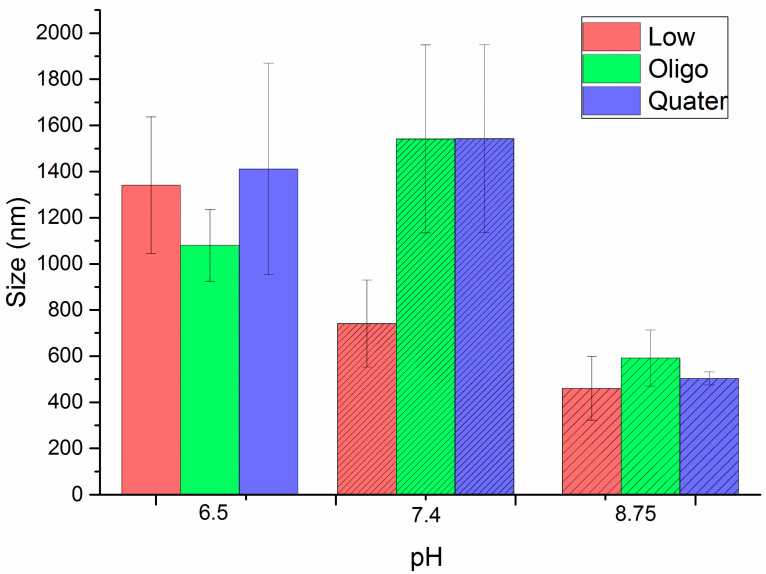
Particle size variation as a function of pH for each of the synthesized nanogels.

In addition, the particle sizes were also determined by DLS after dispersing the synthesized nanoparticles in different media (Figure 8). Three colloidal dispersions were prepared: distilled water (pH = 6.3), distilled water adjusted to physiological pH (pH = 7.4) using a 0.1 M NaOH solution, and the DMEM F:12 PIS culture medium.

Table 2 summarizes the values of the polydispersity indices obtained for the different systems in each medium. Considering monodisperse particles for values less than 0.1, it can be stated that polydisperse swollen nanogels were obtained.

On the other hand, nanoparticles exhibited relatively wide distributions of particle sizes in the swollen state (Figure 8), similar to those previously published by other authors [38,39]. This trend is especially pronounced for biopolymer-based nanogels [40,41]. Taking into account that chitosan is an adhesive polymer when it is in an aqueous medium, chitosan nanoparticles tend to form aggregates, increasing the heterogeneity (polydispersity indices) and size distribution. Nevertheless, aggregation is restricted by using progressive dilution of the samples, which reduces the interaction between particles and, therefore, the formation of large aggregate particles [29].

On the other hand, the swelling (as particle size) of the nanogels in response to the variations in the pH of the media was analyzed from the DLS measurements in the different media. Figure 8 shows the variation in the particle sizes for each nanogel according to the pH of the media, indicating a variable sensitivity of the chitosan nanogels when modifying the pH. The change in the swelling observed in the chitosan nanogels can be considered as a consequence of the electrostatic repulsion forces between the cross-linked polymer chains due to the presence of positive charges.

In this case, the different types of chitosan used had ionizable groups (chitosan and oligosaccharide chitosan) or permanent charges (quaternized chitosan), so their swelling will be conditioned by the repulsive interaction between these groups. Thus, the chitosan and the oligosaccharide presented –NH_2_ groups, whose pKa corresponded to 6.5 [7], which means that at lower pH values, these groups will be ionized as –NH_3_^+^ in such a way that the electrostatic repulsion between these generated positive charges leads to the swelling of the nanogels. In contrast, at higher pH, the swelling should be lower. Indeed, this decrease in the swollen particle size when the pH increases can be clearly observed in Figure 8 for the nanogels prepared from the low molecular weight chitosan. However, when it comes to oligosaccharide chitosan, the modification with PEGBCOOH seems to increase the water dispersibility at neutral pH, regardless of the decrease in the ionization of amine moieties. This fact could be ascribed to the high hydrophilic nature of the introduced PEG, with similar molecular weight than the employed chitosan combined with a reduction in the cross-linking effectiveness derived from the short length of the oligomeric units.

Regarding the quaternized chitosan nanogels, which has a permanent cationic charge, there was no effect of pH when comparing 6.3 and 7.4, and the sizes did not change with pH. However, a reduction in particle size was observed in all cases in the culture medium, at around 8.75. This is due to the effect known as “salting out” or saline precipitation, which is based on the fact that as the ionic strength of the medium increases, the nanogels precipitate due to the neutralization of charges, leading to prevailing hydrophobic interactions against electrostatic repulsions, which gives rise to larger hydrodynamic diameters [42,43].

**Figure 9:**
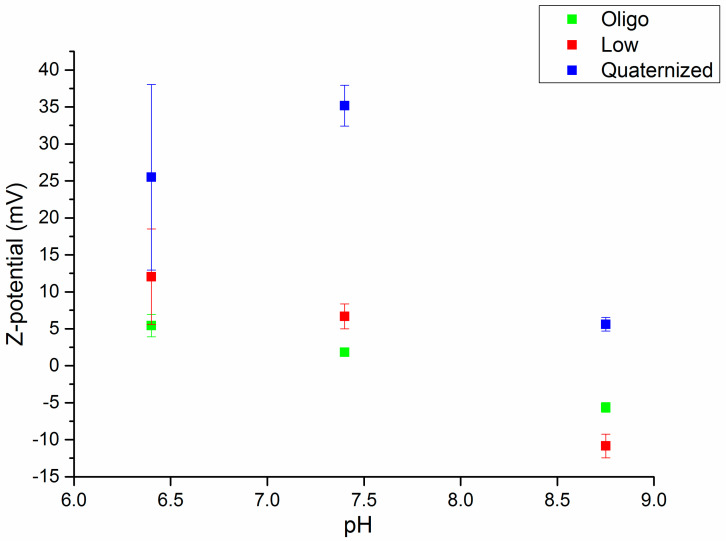
ζ-potential with the pH for each of the synthesized nanogels.

In addition, measurements of the ζ-potential were carried out to analyze the distribution of the surface charge at the nanogel/water interface (Figure 9) and to study the effect observed in the swelling of the nanogels in the different media.

Regarding the ζ-potential measurements, all of the nanogels showed positive potential values at acidic and neutral pH. In the case of low molecular weight and oligosaccharide chitosan, a higher charge was observed at acidic pH than at physiological pH (7.4), which corresponded to a greater ionization of amine groups at pH values lower than 6.5. It should be noted that in the case of quaternized chitosan nanogels, the positive charge was much higher than for these previous cases, in which the cationic charge came from the simple ionization. However, it is worth noting the transformation of the positive surface charge of these hydrogels into negative [(-8)-(-14)] mV when the nanogels were dispersed in the culture medium (pH 8.75). This variation implies the neutralization of the surface electrostatic charge of the nanogels, and therefore the limitation in their swelling, which had already been appreciated in the swelling study.

### 4.4. Nanogels Water Dispersibility

Since chitosan nanogels that are soluble at physiological pH have gained increasing interest in the biological field, the dispersibility of the synthesized chitosan nanogels was evaluated. As mentioned, chitosan is not soluble at neutral or basic pH due to its peculiar conformational characteristics acquired through regular intra- and/or intermolecular interactions [44]. To dissolve chitosan, and subsequently to make its nanogels dispersible, polymer–solvent interactions must be superior to polymer–polymer interactions. At acidic pH, the free amines of the unmodified chitosan chains are protonated, making the polycationic–solvent forces more important than the polymer–polymer, and thus soluble in water [45].

The dispersibility of the chitosan nanoparticles is closely related to the hydrophilic character of the cross-linking agent and to the amount of opposite charge in the chitosan structure. To measure the maximum dispersibility of each of the synthesized nanogels, their dispersions were prepared in three different media: aqueous medium at pH = 6.0, pH = 7.4, and in culture medium.

Cross-linking with PEG, due to its hydrophilic nature, increases the dispersibility of chitosan at physiological pH and gives rise to the formation of fully dispersible nanogels, even at slightly basic pH values.

**Figure 10:**
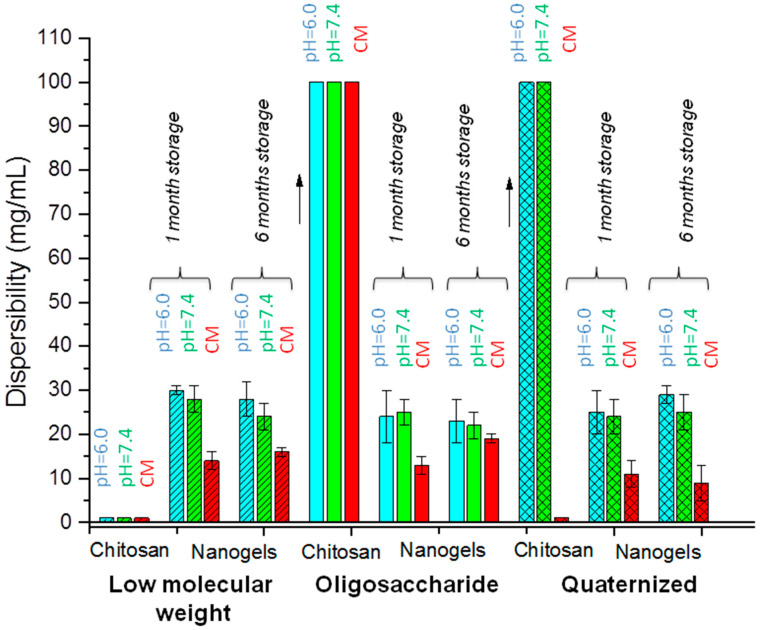
The estimated solubility of the pristine chitosans and the dispersibility of the synthesized nanogels in different media (water pH = 6.0, water pH = 7.4, and culture medium (CM)) for a 1- and 6-month storage period.

Figure 10 shows that, as expected, the soluble chitosans (oligosaccharide in all media, quaternized chitosan at pH = 6.0 and 7.4) gave rise to dispersible nanogels. However, dispersible nanogels could also be obtained from insoluble chitosans by cross-linking with PEGBCOOH. As shown in Figure 10, low molecular weight chitosan nanogels are dispersible even in neutral media, despite initial chitosan insolubility due to the hydrophilic nature of the employed cross-linking agent. In addition, despite its initial insolubility, these nanogels presented a similar dispersibility than the oligosaccharide and quaternized chitosan nanogels. Nevertheless, it seems that the higher surface charge of the quaternized chitosan observed in the zeta potential measurements favors polymer gelation in the culture media, and gives their nanogels a stronger dependence of their stability with the ionic force of the media. This makes a decrease in their dispersibility more noticeable in the culture medium as a consequence of the high electrolyte content. However, the prepared nanogels were dispersible even in the culture media, despite the gelation observed for the initial quaternized chitosan.

Along this line, in all cases, a decrease in the ability to be dispersed in the culture media was observed with respect to water due to the neutralization of the surface charge of the nanogels in this medium. The neutralization of surface charges gives rise to the formation of agglomerates of nanoparticles as a result of greater attractive forces between polymer chains compared to repulsive forces, and therefore to a lower dispersibility in the culture medium.

Despite the markedly lower molecular weight of oligosaccharide chitosan that could limit the hydrophobic interactions, similar dispersibility values were obtained for its nanogels in comparison with the rest of the studied chitosans.

The above described results were corroborated after a 6-month storage period of dried nanogels, which highlights the stabilizing role of the employed cross-linking agent, minimizing the aggregation of the prepared nanogels.

## 5. Conclusions

Chitosan nanogels can be successfully prepared from low molecular weight chitosan, oligosaccharide chitosan, and quaternized chitosan by chemical cross-linking with PEGBCOOH using the inverse microemulsion method. The expected colloidal size of the dried nanoparticles was corroborated by TEM. The particle size of each of the swollen nanogels varied when the pH of the medium was changed as a consequence of the electrostatic repulsions derived from the presence of positive charges in their structure. Furthermore, the zeta potential measurements corroborated the pH sensible swelling behavior of the nanogels according to the electrostatic repulsion between the positively charged particles. In addition, and in accordance with the aim of this work, the incorporation of PEGBCOOH in the structure of the different chitosans greatly increased their dispersibility in all media, which was especially noteworthy for insoluble chitosan. Nevertheless, the formation of agglomerates due to the neutralization of surface charges decreased, to a certain extent, the dispersibility of nanogels in a culture media due to its high electrolyte content. The observed high water dispersibility at physiological pH and in culture media as well as the pH-responsive nature of these biodegradable nanogels make them attractive candidates as nanocarriers for biomedical applications.

## Figures and Tables

**Table 1:** Cross-linking degree of nanogels synthesized from different chitosans and chitosan derivatives estimated as half of the functionalization grade, measured by the Hb and Ha ^1^H NMR relative intensities.

Chitosan	GlcN in Linear CHI (mol. %)	GlcN in CHI Nanogel (mol. %)	Modified –NH_2_ (mol. %)	Cross-Linking (mol. %) ^c^
Low molecular weight	64.0 ^a^	11.6 ^a^	81.9	26.2
Oligosaccharide	85.1 ^a^	0.8 ^a^	99.0	42.2
Quaternized chitosan	3 ^b^	0 ^b^	3	1.5

^a^ determined by ^1^H NMR. ^b^ determined by the ninhydrin test. ^c^ per monomeric unit.

**Table 2:** Polydispersity index values for each of the nanogels at different media.

Chitosan	pH = 6.3	pH 7.4	DMEM F:12 PIS
Low	0.774 ± 0.167	0.595 ± 0.079	0.533 ± 0.135
Oligosaccharide	0.632 ± 0.074	0.747 ± 0.084	0.571 ± 0.069
Quaternized chitosan	0.763 ± 0.173	0.594 ± 0.056	0.483 ± 0.062

## Data Availability

The data generated during this work will be stored at the Figshare repository.
